# Understanding differences in access and use of healthcare between international immigrants to Chile and the Chilean-born: a repeated cross-sectional population-based study in Chile

**DOI:** 10.1186/1475-9276-11-68

**Published:** 2012-11-16

**Authors:** Baltica Cabieses, Helena Tunstall, Kate E Pickett, Jasmine Gideon

**Affiliations:** 1 Faculty of Medicine Universidad del Desarrollo, Avenida Las Condes 12.438 Lo Barnechea, Santiago, Chile; 2 Department of Health Sciences, University of York, Seebohm Rowntree Building, Area 3, York, YO10 5DD, England; 3 University of Edinburgh, Geography Building, Drummond Street, Edinburgh, EH8 9XP, Scotland; 4 London International Development Centre, Birkbeck College, 36 Gordon Square, London, WC1H 0PD, England

**Keywords:** Migration, Healthcare utilization, Access to healthcare, Latin America, Chile, Healthcare inequity, Population-based study, Cross-sectional design, Migración, Utilización de asistencia sanitaria, Acceso a servicios de salud, América Latina, Chile, Inequidad en atención sanitaria, Estudio de base poblacional, Diseño transversal

## Abstract

**Introduction:**

International evidence indicates consistently lower rates of access and use of healthcare by international immigrants. Factors associated with this phenomenon vary significantly depending on the context. Some research into the health of immigrants has been conducted in Latin America, mostly from a qualitative perspective. This population-based study is the first quantitative study to explore healthcare provision entitlement and use of healthcare services by immigrants in Chile and compare them to the Chilean-born.

**Methods:**

Data come from the nationally representative CASEN (Socioeconomic characterization of the population in Chile) surveys, conducted in 2006 and 2009. Self-reported immigrants were compared to the Chilean-born, by demographic characteristics (age, sex, urban/rural, household composition, ethnicity), socioeconomic status (SES: education, household income, contractual status), healthcare provision entitlement (public, private, other, none), and use of primary services. Weighted descriptive, stratified and adjusted regression models were used to analyse factors associated with access to and use of healthcare.

**Results:**

There was an increase in self-reported immigrant status and in household income inequality among immigrants between 2006 and 2009. Over time there was a decrease in the rate of immigrants reporting no healthcare provision and an increase in reporting of private healthcare provision entitlement. Compared to the Chilean-born, immigrants reported higher rates of use of antenatal and gynaecological care, lower use of well-baby care, and no difference in the use of Pap smears or the number of attentions received in the last three months. Immigrants in the bottom income quintile were four times more likely to report no healthcare provision than their equivalent Chilean-born group (with different health needs, i.e. vertical inequity). Disabled immigrants were more likely to have no healthcare provision compared to the disabled Chilean-born (with similar health needs, i.e. horizontal inequity). Factors associated with immigrants’ access to, and use of, healthcare were sex, urban/rural status, education and country of origin.

**Conclusion:**

There were significant associations between SES, and access to and use of healthcare among immigrants in Chile and a higher prevalence of no health care provision entitlement among poor and disabled immigrants compared to the Chilean-born. Changing associations between access and use of healthcare and SES among immigrants in Chile over time may reflect changes in their socio-demographic composition or in the survey methodology between 2006 and 2009.

**Resumen:**

**Introducción**

La evidencia internacional indica menor acceso y uso de servicios de salud por parte de inmigrantes, pero sus factores desencadenantes varían significativamente dependiendo del contexto. Algunas investigaciones se han desarrollado en este tema en América Latina, desde una perspectiva cualitativa. Este estudio cuantitativo exploró el auto-reporte de acceso y uso de servicios de salud de inmigrantes en Chile y los comparó con la población chilena.

**Métodos**

Análisis secundario de datos de encuesta nacional CASEN 2006 y 2009. Inmigrantes fueron comparados con chilenos en características demográficas (edad, sexo, urbano/rural, composición del hogar, etnia), estatus socioeconómico (educación, ingreso, situación contractual), tipo de previsión (pública, privada, otra, ninguna), y uso de varios servicios de atención primaria. Análisis descriptivo, estratificado, y modelos de regresión ponderados para entender factores asociados al acceso y uso de servicios de salud en STATA 11.0.

**Resultados**

Se observó un aumento de reporte de inmigrantes y de la desigualdad en el ingreso de inmigrantes entre 2006 y 2009. Hubo una disminución en la tasa de inmigrantes sin previsión y un aumento en el acceso al sistema privado. Los inmigrantes usaron más frecuentemente la atención prenatal y ginecológica, y menos la atención del niño sano. No hubo diferencia en el uso de citología vaginal o el número total de atenciones recibidas en comparación con los chilenos. Los inmigrantes en el quintil inferior de ingresos reportaron 4 veces más la ausencia de prestación de salud que los chilenos (inequidad vertical). Similares resultados se observaron al comparar inmigrantes con discapacidad con chilenos con discapacidad (inequidad horizontal). Los factores asociados con el acceso y uso del servicio de salud por parte de los inmigrantes fueron el sexo, urbano/rural, educación y país de origen.

**Conclusión**

Se observó una asociación significativa entre estatus socioeconomico, migración y acceso y uso de asistencia sanitaria. Los resultados de este estudio pueden deberse a verdaderos cambios en la composición de los inmigrantes y sus patrones de uso del sistema de salud chileno, pero también pueden deberse a cambios en la metodología y recolección de datos entre encuestas 2006 y 2009. Este aspecto requiere mayor análisis y debate en la región.

## Introduction

Equity in healthcare is of vital importance to many countries. Access to healthcare in particular, has been recognised as a determinant of health and evidence that this is an important issue among immigrants has grown in recent years. This study explores healthcare provision entitlement and use of healthcare services by international immigrants and compared them to the Chilean-born population. This analysis used data from the anonymous national representative CASEN survey in two time points, 2006 and 2009, as they were the first national surveys in Chile to include a set of questions on migration status. Results from this study could inform researchers and policy makers about the most frequent constraints to access and use of the available healthcare system by migrants in this region. It could also inform public health practitioners and global social researchers with an interest in equity in health more generally.

### Migration patterns in Latin America and Chile

Worldwide, it is estimated that 200 million people migrate every year [1,2]. In Latin America and the Caribbean, some 25 million (about 4% of the total population) had migrated to a different country in 2011 [3]. In general, the US is the preferred destination for migrants from Latin American and Caribbean nations [4,5] and income differences between countries are one of the major reasons for these movements [4,6]. There is also increasing migration within the Latin American region, predominantly the movement of people living in relatively less developed countries to more developed ones nearby [2].

Chile is a middle-income country with a Gross Domestic Product per capita of $15 866 (USD) [7]. It has a population of just over 16 million inhabitants and in recent decades has experienced major economic and demographic changes, a progressive improvement of the health status of the population, a decline in the infant and general mortality rates, and an increase in life expectancy [8,9]. Nowadays, the health status of the Chilean population is very similar to some high-income countries and better than many other Latin American nations [10,11]. There are multiple reasons for the relatively good health status of the Chilean population compared to other middle-income countries, which are intimately connected with the economic and social stability of the past century. Since the early 20th century Chile has developed major public health initiatives, firstly focused primarily on maternal-infant mortality and infectious epidemics and more recently on chronic diseases and cancer [12,13]. However, not all socioeconomic groups have benefited from these developments to the same extent [9]. There are significant differences in the health status of Chilean population when comparing by type of healthcare system [14], region [15], gender [16], age [13], and other factors [17,18].

In contrast to other countries in the Latin American region, like Argentina or Brazil, Chile is predominantly a migrant sending country rather than a receiver country. About 857 781 Chileans live outside of the country, with a ratio of 3 Chileans out of country to every immigrant in Chile [2,19]. However, immigration to Chile has increased in recent years, reaching around 1.8% of the total population in 2007 (258–350 thousand persons), which is the highest rate since the 1950s [2]. During the 1970s and 1980s, the emigrant group was composed of political refugees and highly-educated population aiming to improve their living conditions during the military dictatorship. Immigration to Chile was mainly from Europe, Arabic and East Asia until 1982 [20]. During the last two decades, however, South American and other Asian countries have increased their immigration rate [3]. The latest governmental figures indicate that currently Chile is experiencing a “new immigration” pattern with a majority of Latin American immigrants of working age, seeking labour opportunities [2]. There has also been increasing female immigration in the Latin American region, including Chile [3,21], in particular to work in manual and domestic services [22].

### The Chilean healthcare system and the healthcare reform of 2003

Chile is divided into 15 regions and 351 communes or boroughs. The municipality represents the local government agency within the commune. It is responsible for public primary healthcare through primary care centres, which work closely with public hospitals located in their catchment area. The public primary healthcare service in Chile provides some universal services like Pap smears, gynaecological checks, antenatal care, care for people with chronic conditions like diabetes mellitus and hypertension and preventive care for the elderly [23]. If individuals do not want to use the public system, they can choose to attend the private system and pay for these services. Despite the existence of these universal services in the public system, they are not always fully used and there are significant gradients in use by socioeconomic status (SES) in the Chilean population [24].

The Chilean healthcare system has experienced significant changes over time. It was a public system only from its creation in 1952 until the military regime, 1973–1989. During the early 1980s, the military government undertook a series of measures to stimulate growth in membership of the private healthcare system. This reform was based on the principles of “individual freedom, justice (to give each one according to their own contribution), property rights, and subsidiarity” [25]. It understood healthcare as a commodity and created a market for health insurance. Since then, the Chilean healthcare system has been a mixed system characterized by segmentation. With regards to both insurance and supply, public and private sectors coexist with fairly poor interaction between them. The private system covers about 25% of the wealthiest and healthiest population and the public sector covers around 60% of the population, including most of the disabled, sick and elderly. The public system is broadly divided into a 100% ‘free of charge’ service, available to those living below the means-tested poverty line and the ‘public with co-payment’ service that varies in the proportion to be paid according to household earnings. The rest of the population is either part of the Army healthcare system (around 4%) or have no healthcare coverage at all (around 10%) [9,14,26]. With the exception of the public ‘free of charge’ provision that is given to the poorest in the country, everyone can choose between a range of healthcare schemes, both public and private (the latter with over 2500 different schemes available). On top of this, every person can choose to pay for private health insurance, which can come from a Chilean or international private insurer. Less information about this additional health coverage is known in Chile.

During President Ricardo Lago’s term of office (2000–2006), Chile carried out a new healthcare reform that aimed to reduce health inequities by improving the health status of the worst-off social groups. This reform followed the principles of “right to health, equity, solidarity, efficiency, and social participation” [27] and aimed at guaranteeing equal health and healthcare for all Chilean people according to their need and without discrimination of any kind [28]. The healthcare reform was implemented in 2003 and it was expected to produce a significant impact on the population`s health [29], by defining a set of health interventions that, according to the System of Health Guarantee’s Law, should be provided to every person that required them in Chile irrespective of provision entitlement, ability to pay, or any other non-need factor.

Despite these improvements, the Chilean healthcare system does not provide full universal coverage irrespective of migration status. International immigrants with up-to-date legal documentation are entitled to primary healthcare and can choose between the public healthcare system and paying for a private provider. Undocumented immigrants are not allowed to register in the public or private healthcare systems, but could use the private provider for isolated appointments without being registered. A set of specific strategies to support the health of immigrants regardless of their legal status has been recently developed (see Table 1). All immigrants can receive prenatal care, child healthcare, emergency care, and the universal child vaccination programme irrespective of their legal status in the country. However, many immigrants are not aware of these benefits and may still lack preventive and non-emergency care.

**Table 1:** Healthcare programmes recently developed in Chile to protect the health of international immigrants, regardless of their legal status in the country

**Programme**	**Description**
1. *Programme for pregnant immigrant women*	Supported by the Social Organizations Directorate, the Chilean Ministry of Health and the Department of Immigration and Migration, migrant women who are pregnant and have no current legal documentation can attend the primary clinic nearest their home.
2. *Programme for immigrants under 18 years old*	A collaborative agreement between the Chilean Ministry of Health and the Ministry of the Interior. Vulnerable immigrants in this age group can receive healthcare in the public health system, on an equal basis to the Chilean-born regardless of their immigration status and that of their parents.
3. *Free medical care for Peruvians with precarious resources*	Since late August 2002, the General Consulate of Peru in Santiago has had an agreement with the Chilean Red Cross, supported by additional voluntary contributions of Peruvian community physicians, to provide a Free Medical Clinic to Peruvian immigrants in Chile. This service provides primary care to Peruvians, whether documented or not.
4. *Social security agreement between the Republic of Peru and the Republic of Chile*	A convention between the Republics of Peru and Chile ensures the rights of Peruvian immigrants that are pensioners in Chile to receive health benefits equivalent to those of the Chilean-born, such as retirement pensions and social benefits due to disability.

### Purpose of this study and research objectives

While international immigrants are a growing group in Chile, little is known about their healthcare provision entitlement or degree of use of a range of services available in the country. No quantitative study has been completed in Chile assessing health care among international immigrants. This study therefore explores healthcare provision entitlement and use of healthcare services by international immigrants and compares them to the Chilean-born population. We used data from the nationally representative CASEN survey from two time points, 2006 and 2009, as they were the first national surveys in Chile to include questions on migration status.

The main objectives of this study were the following:

1. To identify the rates of healthcare provision entitlement and use of different healthcare services by international immigrants in Chile, and compare them to the Chilean-born.

2. To identify recent changes in healthcare provision entitlement and use of healthcare services among immigrants and to compare them to the Chilean-born.

3. To explore differences in healthcare provision entitlement and use of healthcare services between the poorest immigrants and the poorest Chilean-born (assessment of crude vertical inequity in access and use based on ability to pay).

Three secondary objectives were also considered:

4. To explore differences in healthcare provision entitlement and use of healthcare services between immigrants and the Chilean-born who are disabled (assessment of crude horizontal inequity in access and use, based on equal health need).

5. To explore the socio-demographic factors associated with the type of healthcare provision entitlement among international immigrants and the Chilean-born.

6. To explore the socio-demographic factors associated with the use of a universal service, the Pap smear programme, among international immigrants and the Chilean-born.

## Methods

### Population

This study is a secondary data analysis of the nationally representative CASEN (Caracterizacion Socio-Economica Nacional) survey conducted in Chile. This population-based survey has been carried out every two to three years by the Chilean Ministry of Social Development (former Ministry of Planning) since 1987 and included questions on migration status for the first time in 2006. This study uses data from the most recent 2006 and 2009 CASEN surveys to explore differences in access and use of healthcare services between immigrants and the Chilean-born. The CASEN 2006 was developed with the support of Universidad de Chile, whereas CASEN 2009 was conducted with the support of Universidad Alberto Hurtado.

The CASEN survey employs multistage probabilistic sampling with two phases (county and household) and is stratified by urban/rural area. The sampling frame included all regions in Chile in 2006 and 2009. The inclusion criteria for selection of counties were: (i) All urban counties with > 40 000 inhabitants, (ii) All rural counties irrespective of the number of inhabitants, (iii) A random selection of a small proportion of urban and rural counties with < 40 000 inhabitants. In 2006, 20 hard to reach counties were excluded, from a total of 605 counties in the sampling frame. In 2009, 17 hard to reach counties were excluded, from a total of 602 counties in the sampling frame. Within each county, households were randomly selected from a Census county dataset available at the National Institute of Statistics in Chile. Sampling included people living in transient camps, who represent less than 1% of the total population, however no people living in institutions (i.e. hospitals, prisons) were interviewed [30].

### Sample

The 2006 sample for the analysis consisted of 268 873 people in 73 720 households (44 854 urban and 28 866 rural ones). The 2009 sample consisted of 246 924 people from a random sample of 74 339 households (47 044 urban and 27 295 rural ones). Both surveys represented counties from over 93% of the total Chilean territory [31]. The mean number of households included in the CASEN per region was representative of the total population within each region and also representative of the population in each urban and rural setting from each region in 2006 and 2009 [32,33].

### Data collection

In both years, data collection was via face-to-face interview by trained interviewers, using a validated questionnaire. The preferred respondent was the reported head of household, followed by their spouse or an adult household member. In most cases the head of the household and spouse provided the information about the household. Information was collected on all members of the household, including both adults and children. The response rate for the survey was around 85% in 2006 and 2009 [31].

### Migration status

In both years, the CASEN survey asked: “In which country was your mother living when you were born?” Those who answered “in a different country from Chile” were identified as international immigrants in the analysis (around 1% of the total sample in CASEN in both years). An additional 0.8% preferred not to report their migration status and were excluded from this analysis. They are being analysed separately. Those that reported being born in Chile were included in the Chilean-born comparison group. It should be noted that because of the cross-sectional nature of the CASEN survey the people reporting being an immigrant are likely to be different in 2006 and 2009. As there are not appropriate longitudinal cohort studies in Chile this study examines population trends through the comparison of two cross-sectional datasets.

### Healthcare provision entitlement

This is a multinomial variable with five categories of self-reported healthcare provision entitlement at the time of the interview: no healthcare provision, public free of charge (to those living below the poverty line in Chile), public with some out-of-pocket copayment, private system, army or any other.

### Use of healthcare services

Three broad questions were considered in this study regarding the use of primary healthcare services in Chile:

a. Use of the Pap smear programme was categorized as a binary variable for sexually active women and those between 25–65 years of age. The CASEN included a question on whether eligible women had taken their Pap smear in the past 3 years (yes/no).

b. Mean healthcare attentions received in the last 3 months was treated as a count variable (range 0–36 in 2006 and 0–47 in 2009) of the number of times each participant reported using any healthcare service in the last three months.

c. Type of healthcare service used was assessed among those who reported using the healthcare system in the last three months, further details were collected on the type of programme/service accessed (seven categories, multinomial variable): well-baby care, antenatal care, chronic disease, gynaecologic care, preventive adult/elderly, other type, and don’t remember.

### Demographic factors

These included age (continuous variable and categorised into three broad age-groups: children under 15, working age 15 to 64, elderly over 64), sex (male/female), marital status (single, married/cohabitant, separated/divorced, widow), urban versus rural area (as defined by the National Institute of Statistics at the borough level), and minority ethnicity, defined as belonging to any of the nine legally recognised pre-Hispanic minority ethnic groups in Chile.

### Socioeconomic status (SES)

a. Household income was obtained from the total household income per capita in the past month in Chilean pesos and converted to USD purchasing power parity for 2006 and 2009 (PPP, continuous variable, USD$1 corresponded to 531 Chilean pesos in the 2006 currency and to 510 Chilean pesos in the 2009 currency) [7].

b. Educational level was categorised by the CASEN survey as the highest level achieved for each member of the household (categorical variable): university, secondary, primary, or no education.

c. Employment status (currently active worker) was a binary variable indicating whether the head of the household reported being employed at the time of the interview (yes/no).

d. Contractual status was a binary variable indicating whether the head of the household reported having a formal work contract at the time of the interview (has a formal contract/does not have a formal contract).

e. Type of occupation was classified by self-report of five possible alternative categories: head/manager, self-employed, employee public sector, employee private sector, and domestic service.

### Country of birth

Country of origin was recorded in five categories: Peru, Argentina, Ecuador, Bolivia and Other; reflecting the high prevalence of Latin American immigrants in Chile, the source of over 70% of the total immigrant population in the CASEN survey. A more detailed description of international immigrants by country of origin can be found elsewhere [34].

### Analysis

#### Descriptive and stratified analysis

Descriptive statistics for demographic and socioeconomic conditions of immigrants and the Chilean-born in 2006 and 2009 were reported as means (continuous variables) and proportions (categorical variables) and their 95% confidence intervals. Rates of healthcare provision entitlement and use of various healthcare services were also estimated, with corresponding chi-square and t-tests for independent samples when comparing between immigrants and the Chilean-born. The same tests were used to compare between 2006 and 2009 rates.

#### Exploring crude vertical inequity in access to and use of healthcare

We explored the existence of vertical inequities in access and use of healthcare services, by comparing the rates of healthcare provision entitlement and use of various healthcare programmes in Chile among immigrants and the Chilean-born living in the equivalent poorest income quintile. We obtained chi-square tests for independent samples when comparing between immigrants and the Chilean-born and between years 2006 and 2009.

#### Exploring crude horizontal inequity in access to and use of healthcare

In order to explore the existence of horizontal inequities in access and use of healthcare services, we further estimated the rates of healthcare provision entitlement and use of various healthcare programmes in Chile among immigrants with disability and their Chilean-born counterparts. We obtained chi-square tests for independent samples when comparing between immigrants and the Chilean-born and between years 2006 and 2009.

#### Socio-demographic factors associated with type of healthcare provision entitlement

Adjusted Relative Risk Ratios (RRR, weighted multinomial logistic regressions for multinomial variables) with their 95% confidence intervals (robust standard errors method applied) were estimated to explore the factors associated with type of healthcare provision entitlement among immigrants and the Chilean-born in both years. These models estimated the probability of being entitled to each type of healthcare provision compared to no healthcare provision at all (reference category), adjusted by several covariates of interest (demographics, SES, and migration-related factors). For variables with more than two categories we tested overall statistical significance with an adjusted Wald test (a p-value < 0.05 represented a significant overall association between the variable and the outcome of interest). We also estimated the multinomial goodness-of-fit test for large sample tests with survey design correction (GOF) (F-adjusted mean residual test) [35,36]. A p-value < 0.05 suggests a good fit of the model.

#### Factors associated with use of universal healthcare services: the example of Pap smear

Adjusted Odds Ratios (OR, weighted logistic regressions for binary variables) with their 95% confidence intervals (robust standard errors method applied) were estimated in order to analyse the factors associated with the use of the Pap smear programme in immigrants and the Chilean-born in both years. These models were adjusted by several covariates of interest (demographics, SES, and migration-related factors). We did not estimate regression models for the other measures of use of healthcare due to smaller sample sizes and to prevent the issue of multiple testing with increased risk of spurious associations [37-39]. Again, for variables with more than two categories we tested overall statistical significance with an adjusted Wald test. We used the Archer and Lemeshow goodness of fit test for a logistic regression model fitted using survey sample data (F-adjusted mean residual GOF test) [40]. A p-value above 0.05 suggests good fit of the model (that is, that the *F*-adjusted mean residual GOF test does not indicate any overall model departure from the observed data).

We explored the potential confounding effects of factors related to immigration and health status [41]. Because of the availability of a large number of potential explanatory variables, we used the results of main effect models and the evidence in the research literature to guide testing of multiplicative interaction terms [42] between significant co-variates in each regression model, interactions with age, sex and SES were of particular interest.

The CASEN surveys were obtained after approval from the Ministry of Social Development in Chile through a secured governmental Web page. Data analyses were conducted with the STATA 11.0 and estimations were weighted to take into account the complex multistage sampling strategy of the survey and, therefore, to attain population-based estimates [43].

## Results

### General migration and demographic patterns

There was a significant increase in the proportion of international immigrants living in Chile between 2006 and 2009, rising from 0.96% to 1.24% of the total population (p < 0.05). There was also an increase in the number of people that preferred not to report their migration status over time (missing values, 0.67% in 2006 versus 0.83% in 2009, p > 0.05).

There was a slight increase in the proportion of the immigrant population that were male between 2006 and 2009 (45.20% versus 48.44%, p > 0.05), and a lower proportion of children among the immigrant population in comparison to the Chilean-born in both years. The opposite pattern was observed for the working age group, with a higher proportion among immigrants than the Chilean-born in both years (79.08% and 76.43%, respectively, p < 0.05). Immigrants less frequently reported living in rural areas (6.03% in 2006 and 6.83% in 2009) and more frequently reported being married (45.49% in 2006 and 53.99% in 2009) than the Chilean-born population (Table 2).

**Table 2:** Description of demographic and socioeconomic variables among the Chilean-born and international immigrants in Chile, the CASEN surveys 2006 and 2009

	**CASEN 2006**		**CASEN 2009**	
	**Chilean-born population mean/prevalence (95% CI)**	**Immigrant population mean/prevalence (95%CI)**	**Chilean-born population mean/prevalence (95% CI)**	**Immigrant population mean/prevalence (95% CI)**
***DEMOGRAPHICS***				
Sex: Male^*^	48.66 (48.40-48.94)	45.21 (41.74-48.72)	48.16 (47.86-48.46)	48.44 (44.26-52.64)
Mean age	*X*=32.97 (32.81-33.12)	*X*=33.41 (31.81-35.00)	X=34.22 (34.03-34.41)	X= 33.95 (31.62-36.28)
Mean age men ^**αα**^	*X*= 31.87 (31.69-32.06)	*X*=33.95 (30.84-36.10)	*X*=41.84 (41.43-42.25)	*X*=32.53 (29.38-35.69)
Mean age women ^**αα**^	*X*=33.98 (33.79-34.18)	*X*=32.97 (30.84-35.10)	*X*=44.15 (43.74-44.56)	X=35.28 (32.97-37.60)
Age categories:				
<16^**αα^	25.27 (24.98-25.55)	13.60 (11.29-16.28)	23.90 (23.58-24.22)	14.60 (10.46-20.02)
16-65^**αα^	66.41 (66.12-66.70)	79.08 (75.92-81.93)	65.42 (65.10-65.75)	76.43 (71.46-80.78)
Over 65	8.32 (8.13-8.52)	7.32 (5.33-9.97)	10.68 (10.43-10.93)	8.96 (6.63-12.01)
Zone: Urban^**αα^	87.14 (87.01-87.27)	93.97 (92.58-95.11)	86.32 (85.95-86.68)	93.17 (89.92-95.43)
Rural^**αα^	12.86 (12.59-13.14)	6.03 (4.89-7.42)	13.68 (13.32-14.05)	6.83 (4.47-10.08)
Marital status:				
Single^**αα^	50.57 (50.31-50.84)	45.81 (42.06-49.62)	50.35 (50.05-50.65)	40.25 (34.99-45.74)
Married^**αα^	40.76 (40.46-41.06)	45.49 (41.66-49.36)	40.65 (40.43-40.73)	53.99 (48.66-59.24)
Divorced^**αα**^	4.56 (4.42-4.71)	4.21 (3.06-5.77)	4.58 (4.43-4.57)	2.96 (2.06-4.24)
Widow	4.07 (3.95-4.19)	4.49 (2.89-6.91)	4.42 (4.27-4.57)	2.80 (1.72-4.51)
Belonging to any ethnic minority group	6.55 (6.52-6.80)	5.57 (3.79-8.10)	6.86 (6.59-7.14)	7.10 (5.04-9.91)
***SOCIOECONOMIC CONDITIONS***				
Educational level:				
No education^**αα^	7.39 (7.23-7.55)	2.38 (1.51-3.73)	7.62 (7.43-7.81)	3.12 (1.85-5.43)
Primary School^**αα^	34.68 (34.33-35.03)	18.79 (16.05-21.88)	33.89 (33.51-34.82)	20.05 (14.52-27.03)
High School	29.68 (29.34-30.03)	33.02 (29.39-36.87)	31.37 (30.96-31.78)	30.69 (25.64-36.25)
University level^**αα^	9.86 (9.57-10.15)	27.32 (23.16-31.98)	15.28 (14.84-15.73)	35.79 (29.09-43.09)
Mean total household income per month: USD ^σ^				
Quintile 1 (poorest)^**αα**^	58.46 (57.77-59.15)	56.67 (50.72-62.62)	61.70 (60.98- 62.43)	49.64 (37.65- 61.62)
Quintile 2	107.78 (107.35-108.21)	109.82 (106.31- 113.33)	114.60 (114.22- 114.99)	113.28 (109.95-116.62)
Quintile 3	158.92 (158.39-159.45)	162.31 (157.51-167.11)	163.52 (163.05- 163.46)	159.23 (155.10- 163.36)
Quintile 4	242.77 (241.72-243.82)	244.91 (237.80-252.03)	241.60 (240.71- 242.48)	237.75 (231.59- 244.00)
Quintile 5 (wealthiest)	777.51 (755.85-799.16)	1,303.14 (1,068.16 -1,538.13)	737.85 (698.63- 776.91)	1,217.91 (795.12- 1,693.65)
Currently active worker^**αα**^	57.16 (56.84-57.48)	60.96 (57.06-64.73)	44.87 (44.49-45.25)	58.08 (52.39-63.56)
Type of occupation:				
Head/manager^*αα^	3.10 (2.89-3.32)	5.23 (3.27-8.26)	3.02 (2.75-3.31)	7.72 (3.95-14.57)
Self employed	20.55 (20.05-21.03)	17.15 (14.02-21.64)	20.34 (19.83-20.87)	17.97 (12.91-24.45)
Employee public system	9.76 (9.42-10.11)	6.35 (4.04-9.85)	11.07 (10.06-11.55)	8.28 (4.91-13.44)
Employee private system^**^	60.94 (60.36-61.51)	54.27 (49.10-59.35)	60.78 (60.14-61.42)	54.13 (47.36-60.75)
Employee domestic service^**αα^	5.65 (5.42-5.90)	16.65 (13.40-20.50)	4.79 (4.55-5.04)	11.90 (7.83-17.69)
Has a contract^**αα**^	76.53 (75.96-77.09)	77.79 (73.01-81.93)	23.16 (22.85-23.48)	30.58 (25.51-36.18)

^σ^1USD in 2006=531 Chilean pesos; 1USD in 2009=510 Chilean pesos [7].

^*^ p value < 0.05 when comparing the same categories between the Chilean-born and the immigrant populations in 2006.

^**^ p value < 0.001 when comparing the same categories between the Chilean-born and the immigrant populations in 2006.

^**α**^ p value < 0.05 when comparing the same categories between the Chilean-born and the immigrant populations in 2009.

^**αα**^ p value < 0.001 when comparing the same categories between the Chilean-born and the immigrant populations in 2009.

### Socioeconomic conditions of immigrants compared to the Chilean-born

We found that the SES of the immigrant population in Chile is highly polarised. The gap between the poorest and the wealthiest immigrants in Chile is much wider than the gap observed within the Chilean-born population. There was a 23-fold difference in the mean household income per capita per month between immigrants living in the richest and the poorest quintiles in 2006, which increased to 24.5 in 2009 (top: bottom income quintile ratio, also called the 20:20 ratio). This difference in the Chilean-born was 13-fold in 2006 and 12-fold in 2009. Between 2006 and 2009, there was a small increase in the proportion of immigrants with only primary school education level (from 21.1% in 2006 to 23.1% in 2009) and simultaneously a large increase in the rate of immigrants with University level education (from 27.3% in 2006 to 35.7% in 2009) (Table 2).

### Healthcare provision entitlement among international immigrants and compared to the Chilean-born

There were significant differences in access to healthcare between the immigrants and the Chilean-born. Immigrants reported a 4.3 fold higher rate of people with no healthcare provision than the Chilean-born population in 2009, growing from a 1.8 times difference in 2006 (3.43% in the Chilean-born versus 14.66% in the immigrant population, p < 0.001). In contrast, the Chilean-born were more likely than immigrants to report access to public ‘free of charge’ provision but there was a modest decrease in the difference between the groups, from a 1.9 difference in 2006 to 1.6 in 2009 (p > 0.05) (Table 3).

**Table 3:** Description of healthcare provision entitlement and use of healthcare services by the Chilean-born and international immigrants in Chile, the CASEN surveys 2006 and 2009

	**CASEN 2006**				**CASEN 2009**			
	**Chilean-born Population**		**Immigrant population**		**Chilean-born Population**		**Immigrant population**	
	**Mean/%**	**95% CI**	**Mean/%**	**95% CI**	**Mean/%**	**95% CI**	**Mean/%**	**95% CI**
***HEALTHCARE PROVISION ENTITLEMENT***								
None or don’t know^**αα^	15.37	14.90-15.86	28.10	23.86-32.77	3.43	3.26-3.73	14.66	9.76-21.42
Public 100% free^**αα^	29.39	28.90-29.89	15.27	12.65-18.33	32.66	32.12-33.20	20.68	16.86-25.10
Public with co-payment ^**α^	47.46	46.89-48.03	39.09	34.73-43.63	48.90	48.28-49.52	40.84	33.75-48.33
Private^**αα**^	2.70	2.50-2.91	1.97	0.85-4.48	13.19	12.59-13.80	21.95	16.40-28.74
Other not stated^**αα^	5.08	4.86-5.31	15.57	12.66-19.01	1.77	1.64-1.91	1.88	1.12-3.15
***USE OF HEALTHCARE SERVICES***								
Pap smear	48.50	47.95-49.04	47.28	42.12-52.49	52.33	51.73-52.94	51.61	45.30-57.86
Number of healthcare attentions received in the last 3months	*X*= 2.02	1.99-2.05	*X*=1.97	1.66-2.27	X=1.90	1.85-1.94	X=1.83	1.30-2.35
Type of services:								
Well baby care^**αα^	23.43	22.79-24.07	9.48	5.46-15.94	20.63	19.95-21.33	3.28	1.74-6.10
Antenatal control^**αα^	3.15	2.91-3.42	11.03	6.27-18.68	3.12	2.84-3.43	12.02	5.57-23.36
Chronic disease care	28.99	28.32-29.66	21.75	13.82-32.52	31.72	30.91-32.54	20.54	12.05-32.78
Gynaecologic care^*αα^	9.08	8.67-9.51	16.38	9.42-26.96	7.12	6.68-7.58	24.00	12.03-40.83
Preventive adult/elderly care^**^	16.79	16.21-17.39	28.29	18.56-40.57	16.87	16.14-17.62	19.21	11.06-31.24
Other care	17.84	17.18-18.52	13.07	0.82-20.20	16.61	15.73-7.52	18.78	10.31-31.74
Don’t remember	0.72	0.57-0.92	-	-	1.03	0.88-1.20	1.16	0.26-5.10

^*^ p value < 0.05 when comparing the same categories between the Chilean-born and the immigrant populations in 2006.

^**^ p value < 0.001 when comparing the same categories between the Chilean-born and the immigrant populations in 2006.

^**α**^ p value < 0.05 when comparing the same categories between the Chilean-born and the immigrant populations in 2009.

^**αα**^ p value < 0.001 when comparing the same categories between the Chilean-born and the immigrant populations in 2009.

Patterns of reported health care entitlement changed significantly between surveys in 2006, over a fifth of the immigrant population reported no entitlement to healthcare in Chile, but this declined significantly by 2009 to 14.66%. There was an even greater fall in reported entitlement to “other not stated” type of provision, declining from 15.57% to 1.88% between 2006 and 2009 (p < 0.001).These declining patterns were accompanied by a significant increase in rates of reported access to the private healthcare system (from 1.97% in 2006 to 21.95% in 2009, p < 0.001).

These large changes in reported access to health care entitlements may be in part artifacts of changes by the CASEN survey to the definitions of entitlements rather than substantive changes to health care. In particular, survey respondents may have been discouraged in 2009 from describing their health entitlement as ‘other not stated’ or reporting ‘no health care’ if they were entitled to purchase private care. This hypothesis has been informally mentioned to us by some field coordinators from the 2009 survey, but we have no further evidence to support it. If these changes were substantive this increase in entitlement to the private healthcare sector could be associated with the increase of immigrants in managerial positions and in the top two income quintiles. It could also represent a better understanding of the Chilean healthcare system by immigrants over time and increasing interest in having access to private clinics in the country, a strong proxy of social status in Chilean-born society. This change could also represent increasing proportions of immigrants with no healthcare provision expressing a willingness to pay out-of-pocket for occasional medical consultations to the private system. The ‘other not stated’ category may contain people with international health insurances that expire after some time out of the country of origin and trends in this category may reflect variations in the cost of international health insurances. The Chilean-born population also showed a decrease in the prevalence of people stating that they had no healthcare provision between 2006 and 2009, but this rate was still significantly lower than among the immigrant population.

Further analysis among immigrants with no healthcare provision in 2009 was conducted. This group contained equal numbers of men and women who were mostly of working age (85.53%), married (60.61%), with up to high-school education level (55.63%), followed by University level alone (41.73%), self-employed (42.12%) followed by heads and managers (27.47%) and without a formal job contract (88.16%). In contrast, the Chilean-born with no provision entitlement were mostly men (59.46%), single (57.19%), with up to high school level education (64.04%), working for the private sector (44.64%) followed by self-employed (39.88%) and almost all of them did not have a formal contract (92.76%). In this case, not having a provision entitlement among the Chilean-born appears to be related to significant socioeconomic deprivation (data not shown).

### Use of healthcare services by international immigrants, compared to the Chilean-born

There was no significant difference in the rate of immigrants and the Chilean-born using the Pap smear programme or the number of healthcare services used in the past three months. However, significant differences were observed when looking in detail at the different types of healthcare services included in the CASEN surveys 2006 and 2009. Immigrant women consistently reported higher use of antenatal care (11.03% versus 3.15% in 2006; 12.02% versus 3.12% in 2009, p < 0.001) and gynaecological care (16.30% versus 9.08% in 2006; 24.00% versus 7.12% in 2009, p < 0.001) than the Chilean-born women. However, immigrants reported a lower use of well-baby care than the local population (9.48% versus 23.43% in 2006; 3.28% versus 20.63% in 2009). No significant difference was observed in terms of chronic care or “other” unspecified care service. The use of preventive adult/elderly care was significantly higher among immigrants in the latest survey only (19.21% versus 16.87%, p < 0.001) (Table 3).

### Exploring vertical and horizontal inequity in access to and use of healthcare

According to data from both 2006 and 2009 surveys, immigrants in the poorest income quintile were more likely to be women (54.1%) and of working age than those in the top income quintile. There was a clear gradient of age by income, that is, the lower the income the higher the prevalence of children in these categories. They were also more likely to live in urban areas and showed a higher prevalence of people with a minority ethnic background. Among immigrants in the poorest income quintile almost 60% had only primary level education, over 90% did not have a work contract and around 60% were self-employed. Data regarding occupation indicated a dramatic decline in the prevalence of immigrants with a formal contract between 2006 and 2009, from 77.7% to 30.5%. This pattern of decrease was also visible among the Chilean-born and might represent the effect of the recent international economic crisis. Overall, the measures of SES all suggested that immigrants are becoming a more polarised and unequal group in Chile over time.

**Figure 1:**
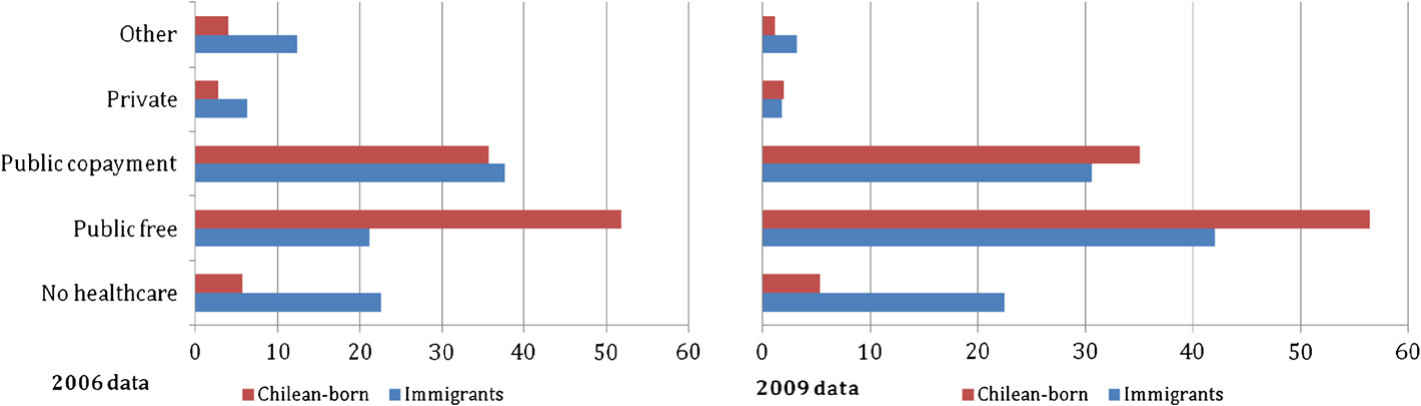
Healthcare provision entitlement of the population living in the poorest income quintile, a comparison between immigrants and Chilean-born, CASEN survey 2006 and 2009.

Exploration of vertical inequity in access to and use of healthcare, comparing immigrants and the Chilean-born living in the poorest household income quintile, indicated that immigrants had about four times higher reported rate of no healthcare provision in 2006 and 2009 than the Chilean-born, but their reported entitlement to public free of charge provision significantly increased in those three years (from 21.13% to 42.0%, p < 0.05), closer to the rates of the poorest Chilean-born. Entitlement to the ‘public with co-payment’ system was not different between the groups under study. Entitlement to the private system fell significantly for this group of immigrants (from 6.24% in 2006 to 1.76% in 2009, p < 0.05) in contrast to the pattern found for immigrants as a whole. The proportion of immigrants in the poorest income quintile reporting “other not stated” healthcare provision also fell from 12.38% in 2006 to 3.17% in 2009 (p < 0.001), getting much closer to the Chilean-born rates (Figure 1).

When exploring healthcare provision entitlement among immigrants and the Chilean-born that reported any disability (crude horizontal inequity), a higher proportion of disabled immigrants reported no healthcare provision compared to the disabled Chilean-born (28.10% versus 15.37% in 2006; 12.72% versus 2.35% in 2009, p < 0.001). There was a lower rate of disabled immigrants entitled to free of charge and with co-payment public healthcare provisions in 2006, and a three times higher rate of “other not stated” provision than among the Chilean-born population that same year. These patterns, however, disappeared in 2009. Regarding the use of healthcare services, similar rates were found to those observed among the total populations, with lower use of well-baby care and higher use of antenatal, gynaecological and preventive adult/elderly care services compared to the Chilean-born population (Table 4).

**Table 4:** Exploration of crude inequality in access to and use of healthcare services among people with heath needs (any disability, either visual, hearing, physical, learning, psychiatric, or speaking), a comparison between the Chilean-born and immigrants, CASEN 2006 and 2009

	**CASEN 2006**				**CASEN 2009**			
	**Chilean-born population with any disability**		**Immigrant population with any disability**		**Chilean-born population with any disability**		**Immigrant population with any disability**	
	**%**	**95% CI**	**%**	**95% CI**	**%**	**95% CI**	**%**	**95% CI**
***TYPE OF HEALTHCARE PROVISION***								
None or don’t know^**αα^	15.37	14.90-15.86	28.10	23.86-32.77	2.35	1.88-2.94	12.72	4.07-33.41
Public 100% free^**^	29.39	28.90-29.89	15.27	12.65-18.33	50.75	49.40-52.10	38.22	21.26-58.62
Public with co-payment^**^	47.46	46.89-48.03	39.09	34.73-43.63	39.99	38.68-41.32	27.36	12.87-48.98
Private	2.70	2.50-2.91	1.97	0.85-4.48	5.29	4.49-6.24	19.29	5.19-51.07
Other not stated^**^	5.08	4.86-5.31	15.57	12.66-19.01	1.61	1.22-1.97	2.41	0.55-9.90
***USE OF HEALTHCARE SERVICES***								
Pap smear	48.50	47.95-49.04	47.28	42.12-52.49	40.81	39.18-42.46	50.99	22.51-78.84
Number of healthcare attentions received	*X*= 2.02	1.99-2.05	*X*=1.97	1.66-2.27	0.70	0.64-0.76	1.07	0.23-1.91
Type of healthcare programme:								
Well baby care ^**^	23.43	22.79-24.07	9.48	5.46-15.94	1.06	0.76-1.47	0	-
Antenatal care^**^	3.15	2.91-3.42	11.03	6.27-18.68	0.74	0.46-1.28	0	-
Chronic disease care	28.99	28.32-29.66	21.75	13.82-32.52	48.80	46.61-50.74	22.66	6.32-59.98
Gynaecologic care^*^	9.08	8.67-9.51	16.38	9.42-26.96	2.42	1.87-3.12	0	-
Preventive adult/elderly care^**αα^	16.79	16.21-17.39	28.29	18.56-40.57	26.19	24.55-27.91	71.72	37.22-91.56
Other attention	17.84	17.18-18.52	13.07	0.82-20.20	18.84	17.05-20.77	6.22	0.88-35.38
Don’t remember	0.72	0.57-0.92	0	-	0.88	0.57-1.35	0	-

^*^ p value < 0.05 when comparing the same categories between the Chilean-born and the immigrant populations in 2006.

^**^ p value < 0.001 when comparing the same categories between the Chilean-born and the immigrant populations in 2006.

^**α**^ p value < 0.05 when comparing the same categories between the Chilean-born and the immigrant populations in 2009.

^**αα**^ p value < 0.001 when comparing the same categories between the Chilean-born and the immigrant populations in 2009.

**Table 5:** Factors associated with use of the Pap smear programme in Chile, a comparison between the Chilean-born and the immigrant populations, the CASEN surveys 2006 and 2009

**Factors**	**CASEN 2006**				**CASEN 2009**			
	Chilean-born population*		**Immigrant population**^**α**^		Chilean-born population**		**Immigrant population**^**αα**^	
	**OR**	**95% CI**	**OR**	**95% CI**	**OR**	**95% CI**	**OR**	**95% CI**
Age	**1.01**	**1.007-1.01**	1.01	0.97-1.06	0.99*	**0.98-0.99**	0.98	0.95-1.01
Marital status:								
Single	1.00	(−)	1.00	(−)	1.00	(−)	1.00	(−)
Married	2.35**	**2.12-2.60**	4.71**	**1.81-12.26**	4.08**	**3.83-4.35**	8.95**	**3.37-23.74**
Divorced	1.77**	**1.53-2.05**	1.94	0.45-8.35	3.22**	**2.90-3.57**	6.22	0.93-41.35
Widow	1.07	0.82-1.40	0.45	0.02-8.82	1.23*	**1.08-1.40**	1.16	0.95-8.87
Zone (rural=1)	1.07	0.93-1.23	4.35	0.80-23.56	1.12*	**1.07-1.18**	2.81**	**1.11-7.12**
Educational level:								
No education	0.47*	**0.29-0.75**	0.48	0.05-4.38	1.70**	**1.52-1.89**	2.48	0.39-15.77
Primary School	0.92	0.77-1.09	0.37	0.11-1.21	1.62**	**1.47-1.82**	2.62	0.45-15.14
High School	0.88	0.76-1.01	0.34	0.11-1.08	1.81**	**1.59-2.07**	2.10	0.35-12.47
University level	1.00	(−)	1.00	-	1.00	(−)	1.00	(−)
Household income, per capita:	1.18	0.97-1.44	0.47	0.08-2.71	1.02*	**1.01-1.03**	0.99	0.98-1.02
Current worker	1.009	0.78-1.30	4.59	0.51-41.12	0.57**	**0.54-0.60**	0.72	0.43-1.52
Has a contract	1.06	0.94-1.18	0.71	0.29-1.73	0.59*	**0.42-0.83**	0.05**	**0.006-0.50**
Peru	-	-	0.99	0.49-2.03	-	-	1.99	0.09-3.03
Argentina	-	-	0.68	0.31-1.47	-	-	1.21	0.61-4.54
Bolivia	-	-	0.42	0.10-1.66	-	-	3.12	0.78-8.58
Ecuador	-	-	1.65	0.46-5.38	-	-	1.65	0.21-7.34
*Armer and Lemeshow GOF test*	*p < 0.001*		*p < 0.001*		*p < 0.001*		*p < 0.001*	

Adjusted weighted logistic regression models, significant co-variates appear in bold in the table, significant overall Wald test for variables with more than 2 categories appear in grey shade in the table. OR: odds ratios, 95% CI: confidence intervals at 95% level.

* p < 0.05.

** p < 0.001.

### Socio-demographic factors associated with healthcare provision entitlement, a comparison between immigrants and the Chilean-born

Significant crude associations between type of healthcare provision and socio-demographics were further explored through adjusted weighted multinomial regressions (baseline category = no healthcare provision). The most parsimonious models for the immigrant and the Chilean-born populations were estimated for 2006 and then repeated for 2009. A detailed table with these results is presented in Additional file 1.

Female immigrants (OR 1.68 in 2006; OR 1.74 in 2009) and immigrants living in rural areas (OR 4.05 in 2006; OR 3.08 in 2009) were significantly more likely to report belonging to the public free of charge provision type. This was also the case for immigrants coming from Argentina (OR 2.88 in 2006; OR 1.44) and Peru (OR 2.39 in 2006; not significant in 2009). There was a clear negative gradient in access to the public free provision by educational level among immigrants in 2006 (Wald test p < 0.001), but it disappeared in 2009. The higher the household income the lower the chance of being entitled to this provision type in 2006 and 2009 data. In contrast, the Chilean-born appeared to have consistent significant associations with age, sex, area type, number of household members, ethnic background, educational level, household income and having a contract. In 2006, among the Chilean-born, women with higher income reported a significantly 35% lower chance of being part of the free of charge public provision (OR 0.65), even after adjusting for sex (OR 1.12) and income (OR 0.99) simultaneously in the model.

Regarding public with co-payment provision type, almost the same patterns as observed for public free provision type was found among immigrants and the Chilean-born in 2006 and 2009. Immigrants had a positive association between this provision type and sex (female OR 1.60 in 2006; OR 1.09 in 2009 [not significant]), area type (OR 2.49 in 2006, OR 1.65 in 2009 [not significant]), and coming from Argentina and Peru (2006 data significant only, OR 2.55 and 2.80, respectively). They also had a negative association with socioeconomic status in 2006, particularly income and education, but this was not observed in 2009.

Different patterns were observed for the private provision type, compared to no healthcare provision. Immigrants showed significant associations with ethnic group (OR 0.15 in 2006; OR 0.10 in 2009, p < 0.001), household income per capita (OR 0.99 in 2006; OR 0.99 in 2009 [not significant]) and coming from Argentina (OR 0.04), Peru (OR 0.58), and Ecuador (OR 0.17) (2006 significant data only). Fewer significant associations were observed in the Chilean-born population in 2006, but 2009 data showed similar patterns to that in the 2006 model.

Immigrants reporting “other not stated” provision type were more likely to live in rural areas (OR 3.06 in 2006; not significant in 2009) and less likely to have higher household income (OR 0.99 in 2006, not significant in 2009) and to come from Ecuador (OR 0.20 in 2006, not significant in 2009), compared to immigrants with no healthcare provision. Similar patterns to the private provision type were found for the Chilean-born population, both in 2006 and 2009. The overall goodness of fit of the models was adequate, especially for the large Chilean-born population.

### Factors associated with the use of the Pap smear program, a comparison between immigrants and the Chilean-born

The few factors that appeared to be significantly associated with the use of the Pap smear programme among immigrants, were being married (OR 4.71 in 2006, OR 8.95 in 2009; Wald test for marital status not significant in both years) and having a work contract (OR 0.05 in 2009, data not significant in 2006). The Chilean-born population in contrast, appeared to have significant associations with age, marital status, rural/urban area, educational level, household income, employment status, and contractual status, both in 2006 and 2009. Goodness of fit tests of the models, however, showed poor fit and therefore further relevant unmeasured variables should be added in this type of analysis in the future, in order to better understand the factors that might determine the use of the Pap smear programme among immigrants and the Chilean-born in the country, the region and more generally (Table 5).

## Discussion

Migration status is a social determinant of health that is intimately associated with healthcare provision entitlement in Chile and Latin America. Access to healthcare is the result of a complex group of determinants [44]. It depends on the extent to which a society is able to create an environment that supports immigrants to overcome the socioeconomic and the cultural or psychological barriers that may limit people's ability to receive care [45,46]. This study finds evidence that some international immigrants in Chile continue to be exposed to socioeconomic risks for health and, even after the equity-centred healthcare reform of 2003, experience limitations to adequate use of healthcare services. The associations between migration status, SES and access to healthcare were substantial. Distinctive patterns in entitlement to different healthcare provisions and the use of primary care services were found among immigrants by SES in this study.

This study indicated an increase in self-reported immigration status in Chile between 2006 and 2009, an increase in the proportion of male immigrants and an increase in income inequality between extreme quintile groups among immigrants. There was a decrease in the rate of immigrants reporting no healthcare provision over time, and an increase in reporting of private healthcare provision entitlement. In contrast to what might have been expected, the international immigrant population reported higher rates of use of several primary services and no difference in the use of the Pap smear programme or the number of attentions received in the last three months than the Chilean-born. They reported, however, a lower use of well-baby care. It is possible that a lack of such services in the countries from which migrants originate mean that they are less likely to make use of these services when in Chile. It is also possible that pregnant immigrant women prefer to return home to deliver their babies and therefore use less well-baby care than might be expected.

Significant differences in healthcare provision entitlement were found between the immigrant and Chilean-born populations living in the poorest income quintiles. Immigrants in the bottom income quintile were around four times more likely to report no healthcare provision than the Chilean-born and this was consistent across 2006 and 2009 surveys. Disabled immigrants were also more likely to have no healthcare provision compared to the disabled Chilean-born. In addition, a range of socio-demographic factors were associated with the type of healthcare provision immigrants were entitled to, including sex, urban/rural status, education and country of origin. Factors associated with the use of the Pap smear programme service among immigrants were marital status and contractual status.

There was no increase in the proportion of female immigrants coming to Chile over time. Female immigrants, however, continue to experience significant limitations to their access to healthcare. They were more likely to be entitled to the public free of charge provision type than men, which is by definition a measure of absolute poverty. A large proportion of immigrants in the poorest income quintile were women and almost all immigrants working in domestic services were women. Even though immigrant women seemed to use most primary care services at similar rates to Chilean-born women, they appeared to consistently use the well-baby care programme less frequently, despite reporting about four times more use of antenatal care than the Chilean-born.

There is a growing interest in the migrant female population worldwide and in the region, as their vulnerability to socioeconomic deprivation, discrimination and ill-health has been confirmed on several occasions [47,48]. There are some studies of the deskilling effects of migration among women in Latin America, for example Bolivians in Argentina [49] and Latin migrants in London [50]. Further analysis needs to be conducted to disentangle the complex relationship between gender, migration, SES and access to and use of healthcare services in Chile and the Latin American region, and how they then relate to health status and perceived wellbeing. This study provides the first repeated cross-sectional analysis on access to and use of healthcare among immigrants in Chile using population-based estimations, and could be expanded to understand further gender issues in the future.

One of the most salient findings from this study was the close relationship between SES and access to and use of healthcare among immigrants in Chile, a similar pattern was also found for the Chilean-born population. Household income, educational level and contractual status were the most prominent dimensions of SES associated with the outcome variables of interest. Educational level was a significant factor associated with healthcare provision entitlement and use of healthcare services in this study. Proposed explanations of the association between greater education and use of healthcare are likely to include better health knowledge and better ability to navigate the healthcare system. Those with higher educational attainment may also have more social contact with physicians, both in university and afterward, than those with lower educational attainment [51]. Preferences and expectations play an important role in accounting for the variation in the use of specialist services between those in high and low social position [52,53]. In this sense, the less educated or poor may be less able to express their need for care [54]. In addition, those in higher social positions may have different attitudes about the benefits that can be realised by accessing care and so may be more motivated to seek opportunities. It is possible then, as stated by Dunlop et al. [52], that those with higher SES can access and thereby benefit more effectively from the health care system than those of low SES.

Although people who migrate are often healthier than native-born residents because of the various selection processes they face, migrants are usually exposed to health risks. Moving to an unfamiliar environment does affect access to healthcare services [55,56]. A growing body of literature indicates that immigrants face individual, socio-cultural, economic, administrative, and political barriers when using health services [57-59]. These can be formal barriers (language, geographical distance, complexity of the structure and processes of the healthcare system) or informal ones (less tangible barriers like perceived discrimination) [60,61]. Providers' attitudes have also been reported as perceived barriers by immigrants and studies have pointed out that stereotypes about migrants' health held by providers often stand in the way of providing the best quality care [57,62,63]. Immigrants, on the other hand, may have different expectations of health and perceptions of appropriate care, based on experiences with the health system in their country of origin [64]. Most of these factors have been reported among Latin American migrants in the region and elsewhere. Such evidence comes predominantly from small qualitative studies and this study complements those data with population-based estimates of relevant demographic and socioeconomic factors associated with access to and use of healthcare by immigrants in Chile.

Findings from this study need to be interpreted cautiously in at least two key aspects. First, we have compared two separate surveys, the CASEN 2006 and 2009. These datasets allow us to explore general migration patterns at different time points, but not to follow the same individuals through this period of time as longitudinal analysis would do. Therefore, we cannot assume that changes in patterns between 2006 and 2009 represent changes within the same individuals, but only to the populations under study as a whole. Second, those that preferred not to report their migration status in both surveys were excluded from this analysis. They might represent undocumented immigrants in fear of prosecution and, hence, might experience very different patterns of access to and use of the healthcare system [65-67]. 

According to Dias et al. [57], understanding the issues related to migrants' health and their utilization of healthcare services is challenging because of gaps in databases, heterogeneity of immigrant populations, and uncertainty about how migration affects health. This study has important strengths but also some limitations. Due to the cross-sectional nature of this study, we cannot determine whether migration is a cause of poor access to some healthcare provision or well-baby care. Nonetheless, the causal relationship between migration and access to healthcare has been considered extensively in past decades, and some evidence suggests a link between them [68,69]. There is also the risk of self-report bias in this study, not only in relation to migration status, but also SES and other measures. Although some limitations of these measures have been recognized in recent decades, they are considered robust measures and are widely used in health research [70]. Findings from this study cannot be extrapolated to the 15% of the population that did not respond to the CASEN survey. Issues related to recruiting hard to reach populations, including undocumented immigrants, will need to be considered by this survey in the future [71,72]. Also, we do not have information on second generation immigrants. For that reason, acculturation processes across generations of immigrants will not be captured in this study, as second generation immigrants might experience larger acculturation effects than first generation ones. This in turn might create diverging health-risk factors that will need to be taken into account separately when such data is available.

The relationship between SES and healthcare provision entitlement among immigrants appears to be significant, however, other factors should also be included in analysis in the future in order to better understand this link, such as legal status, health status, stigma and discrimination, and others [52,57,73-75]. The Chilean health care system does not provide full universal coverage irrespective of migration status. This is a major issue to tackle in the country, especially for well-baby care that showed very low use rates among immigrants, but it is not likely to be sufficient to solve inequity in healthcare. International research suggests that the introduction of universal coverage better supports the distribution of healthcare services according to need, but it does not solve inequity and remove socioeconomic gradients in use [73]. Glazier et al. [51] found that universal health insurance appears to be successful in achieving income equity in primary care, but not in specialist care. Moreover, even in countries where access to health care is guaranteed, immigrants do not always take full advantage of services available [57,59,63]. Equal access for equal need presumes that individuals are given equal opportunities to access services. Inequity in utilization may not solely reflect inappropriate or unfair differences in service use, as utilization is affected by personal characteristics such as individual preferences, expectations and beliefs, and past experiences of stigma and discrimination [76,77]. There is much more to untangle, understand and improve in this field.

## Conclusion

This is the first quantitative study to explore healthcare provision entitlement and use of healthcare services by international immigrants and compare them to the Chilean-born population. Results represent novel evidence identifying significant diversity and vulnerability among migrants in this region. Future research could disentangle the degree to which changes observed over time represent variations among international immigrants to Chile or are an artifact of changes in the survey data collection techniques.

## Abbreviations

SES: Socioeconomic status; GOF: Goodness of fit.

## Competing interests

The authors declare that they have no competing interest.

## Authors’ contributions

The four authors made substantial contributions to conception and design, analysis and interpretation of data, drafting of the manuscript, and final approval of the version to be published. BC developed the study design, conducted the analysis and drafted the manuscript. HT participated in the design, analysis, interpretation and drafting of this paper. KP participated in the design, analysis, interpretation and drafting of this paper. JG participated in the design, interpretation and drafting of this paper. All authors read and approved the final manuscript.

## Supplementary Material

Additional file 1 Factors associated with provision entitlement in Chile, a comparison between the immigrant and the Chilean-born populations, the CASEN surveys 2006 and 2009. Click here for file
