# Supplementary material for: Understanding differences in access and use of healthcare between international immigrants to Chile and the Chilean-born: a repeated cross-sectional population-based study in Chile
Source: Int J Equity Health. 2012 Nov 16;11:68. doi: 10.1186/1475-9276-11-68 (PMC3541972; doi:10.1186/1475-9276-11-68)
Supplement: Additional file 1 — Factors associated with provision entitlement in Chile, a comparison between the immigrant and the Chilean-born populations, the CASEN surveys 2006 and 2009. [file 1475-9276-11-68-S1.docx]

**Additional File 1** Factors associated with provision entitlement in Chile, a comparison between the immigrant and the Chilean-born populations, the CASEN surveys 2006 and 2009

| **Factors** | **CASEN 2006** | | | | **CASEN 2009** | | | |
| --- | --- | --- | --- | --- | --- | --- | --- | --- |
|  | **Chilean-born Population** | | **Immigrant population** | | **Chilean-born Population** | | **Immigrant population** | |
|  | **RRR** | **95% CI** | **RRR** | **95% CI** | **RRR** | **95% CI** | **RRR** | **95% CI** |
| ***FREE PUBLIC HEALTH CARE PROVISION*** | | | | | | | | |
| Age | **0.87*** | **0.83-0.91** | - | - | **0.96*** | **0.95-0.98** | - | - |
| Age2 | **1.001*** | **1.0009-1.002** | - | - | **1.004*** | **1.002-1.006** | - | - |
| Sex (female=1) | **1.12*** | **1.03-1.24** | **1.68*** | **1.03-2.72** | **1.72**** | **1.56-1.94** | **1.74*** | **1.04-2.92** |
| Zone | **2.13**** | **1.73-2.61** | **4.05**** | **1.90-8.60** | **1.61**** | **1.39-1.86** | **3.08**** | **1.19-7.93** |
| Number household members | 0.98 | 0.93-1.04 | - | - | **0.83*** | **0.79-0.87** | - | - |
| Belong to ethnic minority group | **2.02**** | **1.34-3.10** | 2.13 | 0.63-7.14 | **1.59**** | **1.25-2.02** | 1.09 | 0.38-3.52 |
| Education level: |  |  |  |  |  |  |  |  |
| No education | **13.91**** | **1.37-17.47** | **8.24**** | **3.23-12.78** | 1.10 | 0.81-1.50 | **5.86**** | **1.81-18.54** |
| Primary level | **5.36**** | **2.56-7.92** | **4.06**** | **1.56-10.53** | **0.58**** | **0.43-0.80** | 2.30 | 0.88-6.21 |
| High school | **3.90**** | **2.51-6.15** | **3.47**** | **1.36-8.85** | **0.35**** | **0.25-0.48** | 2.42 | 0.75-7.75 |
| University | 1.00 | (-) | 1.00 | (-) | 1.00 | (-) | 1.00 | (-) |
| Household income per capita (continuous) | **0.99*** | **0.99-0.99** | **0.99*** | **0.99-0.99** | **0.98*** | **0.97-0.99** | **0.99*** | **0.99-0.99** |
| Has a contract | **0.31**** | **0.25-0.39** | - | - | **2.03**** | **1.21-3.39** | - | - |
| Argentina | - | - | **2.88**** | **1.47-5.65** | - | - | **1.44*** | **1.02-4.12** |
| Peru | - | - | **2.39**** | **1.03-5.53** | - | - | 0.98 | 0.51-5.74 |
| Ecuador | - | - | 0.35 | 0.05-2.47 | - | - | 0.12 | 0.05-5.28 |
| Interaction female* income | **0.65**** | **0.62-0.69** | - | - | - | - | - | - |
| ***PUBLIC WITH CO-PAYMENT HEALTH CARE PROVISION*** | | | | | | | | |
| Age | **0.90*** | **0.86-0.93** | - | - | **0.93** | **0.91-0.94** | - | - |
| Age2 | **1.001*** | **1.0007-1.002** | - | - | **1.008** | **1.006-1.01** | - | - |
| Sex (female=1) | **1.10*** | **1.001-1.19** | **1.60*** | **1.06-2.41** | **1.60** | **1.43-1.80** | 1.09 | 0.68-1.73 |
| Zone | **1.65**** | **1.38-1.97** | **2.49**** | **1.33-4.67** | 0.93 | 0.80-1.08 | 1.65 | 0.59-4.60 |
| Number household members | 1.001 | 0.96-1.04 | - | - | **0.82*** | **0.78-0.86** | - | - |
| Belong to ethnic minority group | **1.57**** | **1.06-2.30** | 1.53 | 0.47-4.97 | 1.01 | 0.79-1.30 | 0.61 | 0.18-2.04 |
| Education level: |  |  |  |  |  |  |  |  |
| No education | **14.66**** | **6.10-26.12** | **4.57**** | **1.24-16.89** | **2.06**** | **1.50-2.83** | 2.44 | 0.70-8.40 |
| Primary level | **15.24**** | **10.78-21.52** | **2.50*** | **1.03-6.06** | **1.60**** | **1.17-2.20** | 1.59 | 0.44-5.64 |
| High school | **4.92**** | **4.08-5.95** | **3.04**** | **1.33-6.97** | 1.28 | 0.93-1.76 | 7.71 | 0.42-6.92 |
| University | 1.00 | (-) | 1.00 | (-) | 1.00 | (-) | 1.00 | (-) |
| Household income per capita (continuous) | **0.99*** | **0.99-0.99** | **0.99*** | **0.99-0.99** | 0.99 | 0.98-1.01 | 0.99 | 0.98-1.02 |
| Has a contract | **1.60**** | **1.31-1.95** | - | - | **0.48**** | **0.30-0.76** | - | - |
| Argentina | - | - | **2.55**** | **1.38-4.75** | - | - | 0.95 | 0.21-3.21 |
| Peru | - | - | **2.80**** | **1.38-5.90** | - | - | 1.95 | 0.41-4.57 |
| Ecuador | - | - | 0.65 | 0.20-2.07 | - | - | 1.02 | 0.47-5.48 |
| Interaction sex*household income | **0.78*** | **0.74-0.82** | - | - | - | - | - | - |
| ***PRIVATE HEALTH CARE PROVISION*** | | | | | | | | |
| Age | 0.93 | 0.83-1.02 | - | - | **0.90*** | **0.88-0.93** | - | - |
| Age2 | 1.001 | 0.99-1.002 | - | - | **1.008*** | **1.005-1.01** | - | - |
| Sex (female=1) | 1.01 | 0.95-1.12 | 2.62 | 0.62-11.03 | **1.72**** | **1.46-2.02** | 1.30 | 0.62-2.74 |
| Zone | **0.24**** | **0.10-0.56** | 0.45 | 0.04-4.46 | **0.34**** | **0.27-0.44** | 0.56 | 0.18-1.76 |
| Number household members | 1.10 | 0.99-1.22 | - | - | **0.87*** | **0.82-0.93** | - | - |
| Belong to ethnic minority group | 1.24 | 0.36-4.30 | **0.15**** | **0.16-0.78** | **0.46**** | **0.31-0.68** | **0.10**** | **0.01-0.75** |
| Education level: |  |  |  |  |  |  |  |  |
| No education | 10.49 | 0.70-15.62 | **0.52**** | **0.08-0.93** | **4.36**** | **2.21-8.60** | 4.89 | 0.43-15.68 |
| Primary level | 2.34 | 0.67-8.09 | 6.93 | 0.45-10.52 | **5.73**** | **2.88-11.41** | 5.40 | 0.58-10.00 |
| High school | **3.20**** | **1.83-5.60** | 1.51 | 0.12-18.16 | **7.18**** | **3.55-14.64** | **3.78**** | **1.01-15.93** |
| University | 1.00 | (-) | 1.00 | (-) | 1.00 | (-) | 1.00 | (-) |
| Household income per capita (continuous) | **0.99*** | **0.99-0.99** | **0.99*** | **0.99-0.99** | **1.02*** | **1.01-1.03** | 0.99 | 0.98-1.05 |
| Has a contract | **0.30**** | **0.18-0.48** | - | - | 0.64 | 0.38-1.23 | - | - |
| Argentina | - | - | **0.04**** | **0.006-0.29** | - | - | 0.40 | 0.06-2.35 |
| Peru | - | - | **0.58*** | **0.17-0.98** | - | - | 0.62 | 0.27-3.97 |
| Ecuador | - | - | **0.17**** | **0.03-0.77** | - | - | 0.01 | 0.001-2.12 |
| Interaction sex*household income | 1.01 | 0.88-1.16 | - | - | - | - | - | - |
| ***OTHER NOT STATED HEALTH CARE PROVISION*** | | | | | | | | |
| Age | 0.90 | 0.83-0.98 | - | - | **0.94*** | **0.92-0.96** | - | - |
| Age2 | 1.001 | 0.99-1.002 | - | - | **1.008*** | **1.005-1.01** | - | - |
| Sex (female=1) | 1.07 | 1.001-1.17 | 0.94 | 0.59-1.50 | **1.49**** | **1.26-1.72** | **0.36*** | **0.13-0.97** |
| Zone | **1.84**** | **1.35-2.51** | **3.06**** | **1.38-6.78** | **0.42**** | **0.33-0.55** | 0.50 | 0.08-3.11 |
| Number household members | 1.004 | 0.92-1.09 | - | - | **0.82**** | **0.76-0.89** | - | - |
| Belong to ethnic minority group | 1.56 | 0.88-2.76 | 2.85 | 0.95-4.50 | 0.75 | 0.48-1.19 | **0.13*** | **0.01-0.92** |
| Education level: |  |  |  |  |  |  |  |  |
| No education | 4.26 | 0.45-9.90 | 2.21 | 0.54-9.04 | **3.26**** | **1.73-6.15** | 2.33 | 0.48-4.45 |
| Primary level | **8.28**** | **4.73-14.50** | **2.09**** | **1.007-4.36** | **5.37**** | **2.86-10.09** | 1.60 | 0.10-2.47 |
| High school | **2.91**** | **1.86-4.54** | 1.57 | 0.79-3.12 | **3.92**** | **2.06-7.43** | 1.88 | 0.65-11.81 |
| University | 1.00 | (-) | 1.00 | (-) | 1.00 | (-) | 1.00 | (-) |
| Household income per capita (continuous) | **0.99*** | **0.99-0.99** | **0.99*** | **0.99-0.99** | **1.02*** | **1.01-1.03** | 0.99 | 0.98-1.00 |
| Has a contract | **0.17**** | **0.12-0.25** | - | - | 1.47 | 0.74-2.92 | - | - |
| Argentina | - | - | 0.95 | 0.48-1.89 | - | - | 0.23 | 0.05-2.47 |
| Peru | - | - | 1.50 | 0.65-3.48 | - | - | 0.94 | 0.29-3.50 |
| Ecuador | - | - | **0.20**** | **0.06-0.68** | - | - | 1.85 | 0.17-5.98 |
| Interaction sex*household income | **0.79**** | **0.73-0.85** | - | - | - | - | - | - |
| *Pearson´s Chi square GOF test (Jann, 2008)* | *p<0.001* | | *p<0.001* | | *p<0.001* | | *p>0.05* | |

Adjusted weighted multinomial logistic regression models (no healthcare provision reference category). Significant co-variates appear in bold font in the table, significant overall Wald test for variables with more than 2 categories appear in grey shade in the table. RRR: relative risk ratios, 95%CI: confidence intervals at 95% level.

* p value <0.05

** p value <0.001
